# The Institutional Library

**Published:** 1916-03-04

**Authors:** 


### THE INSTITUTIONAL LIBRARY.

### EVOLUTION AND THE WAR.

**Evolution and the War.** By P. Chalmers Mitchell, M.A., D.Sc., etc. (London: John Murray. 1915. 2s. 6d. net.)

"The scientific spirit" is a much-abused phrase, that is used glibly by many who attach to it no very definite meaning, but he who would understand what it ought to mean could scarcely find a better example in which to study it than this book of Dr. Chalmers Mitchell's. In it the reader will find words used strictly and accurately in their proper meanings; he will find facts distinguished clearly from inferences; and he will find, what is so rare to find, an hypothesis, however alluring, however satisfying, however fully supported by facts, such, for instance, as the hypothesis of Natural Selection, still treated as an hypothesis, still regarded with an open mind, still looked upon as possibly erroneous and supersedable, if, in spite of its plausibility, it is not fully entitled to be considered an established law. It is in these things that the scientific spirit consists.

The Germans, who justify and advocate war as the noblest occupation of man, do so very largely on the ground that war is but one exemplification of "the natural law of the struggle for existence," a law which holds good throughout the whole world of living beings, and is as true of nations as of species. Dr. Chalmers Mitchell proves conclusively that this doctrine is founded upon ignorance of what is meant by a natural law, on ignorance of what is meant by the struggle for existence, and on an unproven assumption that what is true (if it is true) of species of animals and plants is true of nations of mankind also. The "struggle for existence" is not a natural law, it is an hypothesis, very probable, but as yet unproved. Moreover, "the struggle for existence" does not mean war, nor anything resembling war. It is a struggle, not of individual against individual, not of community against community, not of species against species, but of individual, of community, and of species against all those forces and conditions which are destructive, dangerous, or detrimental. The conditions which conduce to success or failure in the "struggle" for life are many of them so elusive, so fine, so subtle, that they cannot be identified; in many cases they cannot be conjectured. When one species replaces another in a certain locality it is not because the members of the prevailing species attack the members of the other species and exterminate them, but because the members of the prevailing species have some advantage, often so slight that we cannot identify it, in variety of diet, or in means of gaining food, or in care of their young, or in ability to resist climate or disease. In many cases the survival value consists not in bellicosity or ferocity, but in sympathy and mutual assistance. It is this last quality which contributes so powerfully to the survival of the Scottish race, an instance which Dr. Chalmers Mitchell unaccountably forgets to adduce.

Moreover, a nation has no similarity to a species; and if it were the case, which it is not, that the struggle for life is a struggle of organisms against one another, and if it were a fact, which it is not, that natural selection is a proven law, still it would not follow that there is any biological necessity or desirability for nations to exterminate one another. A species is a race. All its members are akin in blood. All are blood relatives, and own a common ancestor. All are of similar biological and physiological constitution. A nation is nothing of the sort. It may be, and almost always is, compounded of very diverse ethnic elements. The bond of union of a nation is not in a common blood-relationship, but in a common language, common ideals, a common literature, common aims; and these are the results of a common "Kultur"—that is to say, the influence of similar education, similar social structure, similar customs, laws, habits, and so forth. The member of a species is the material offspring of his progenitors and his ancestry: the citizen of a nation is the mental and moral result of the history of his nation. The one is the product of nature, the other is the product of nurture. If the German is immoral and a brute, it is not because he comes of a different race from the Englishman, the Frenchman, and the Russian, for there are strains of blood common to them all, and the great bulk of the "kultured" German race is of the same blood as the "barbarian" Russian. The German is immoral and a brute because he has been educated to be so, because he has grown up amongst immoral brutes, because immorality and brutality have been held up to him from infancy as proper and desirable; because, in short, he comes of the German nation, not of any German race, for there is a German nation, but there is no German race.

**Training in First Aid and Nursing by Question and Answer.** By Captain S. T. Beggs, M.D. (London: The Scientific Press. Price 1s. 6d. net.)

This catechism comprises 600 questions and answers on first aid and elementary nursing. This method of teaching elementary students in all branches of education is too well known to need description; and the absolute trustworthiness of the answers is attested by the fact that they are derived, by permission, from the Training Manual of the Royal Army Medical Corps. The questions are judiciously framed, and altogether the book should fill a distinct place in the special literature of V.A.D. and ambulance detachments.

**Tuberculosis.** A General Account of the Disease. Its Forms, Treatment, and Prevention. By A. J. Jex-Blake, M.D., F.R.C.P. (London: G. Bell and Sons, Ltd. 1915. Price 2s. 6d.)

In these days the man in the street is provided in one way and another with a good deal of educative pabulum on the subject of tuberculosis. Much of this is doubtless regarded by the more intelligent and better-instructed layman as being restricted in scope and more or less self-evident. It is therefore a matter for congratulation that there is now available at a moderate price a book which goes much more thoroughly and more deeply into the whole subject of tuberculous disease. Dr. Jex-Blake in well-arranged and skilfully written chapters considers thoughtfully the doings of the tubercle bacillus and the ways and means by which it may be defeated or at least discouraged. Starting with a brief survey of the history of medicine as related to tuberculosis, the author boldly enters into a discussion of the pathology of the disease. It is here, perhaps, that he has found most difficulty in avoiding the use of highly technical terms; and though it would be too much to say that the non-professional reader will readily comprehend all the details, yet it must be admitted that the account, for example, of the paths of infection is a very creditable performance. This chapter is one which, if read with care, cannot fail to be of interest and profit to the reader. But the same may be said of nearly all the pages of the book, on which are described in turn diagnosis, prognosis, treatment, general and specific, and prevention. It may be questioned whether the inclusion of many highly technical procedures in connection with the diagnosis of tuberculosis and its specific treatment with various tuberculins, nascent iodine, or pneumothorax was desirable in a book of this character; but, having regard to the cautious attitude maintained and the healthy scepticism imported into the subject-matter, their inclusion will probably do good. Sanatorium treatment is well described, and Dr. Jex-Blake uses the words in the original sense as meaning treatment at a sanatorium. He deplores the misleading usage of the term which is now so prevalent since the passing of the National Insurance Act. It is very interesting to note that the author inclines to the view that a mild infection in early life with bovine tubercle bacilli in milk possibly is an advantage, inasmuch as this may give a degree of acquired immunity which will protect the adult against infection with the more virulent bacilli of human type in later years. This book is really a compendium of the medical and sociological problems of tuberculosis, and as it is obviously authoritative and reliable in the present state of our knowledge it is a work which could with advantage be widely circulated.

**Rural Sanitation in the Tropics.** By Malcolm Watson, M.B. (London: John Murray. 1915. Pp. 320. Price 12s. net.)

The author of this book was fortunate in having passed through the London School of Tropical Medicine just about the time that Sir Ronald Ross's discoveries and their confirmation by Manson and others had opened the eyes of tropical hygienists to the possibilities of controlling that chiefest of tropical scourges, malaria. Thoroughly imbued with Ross's doctrines, and enthusiastic to put them to the test, Dr. Watson opportunely obtained an appointment in the Federated Malay States just when the opening up of that virgin country was leading to appalling malaria statistics and the threat of ruin instead of the brilliant future which had seemed to be in store. Fortunately, too, he is not merely an indefatigable worker, but a man capable of looking new facts in the face and of adapting his foundation principles to meet new problems. Having first scored a tremendous success in eradicating malaria from the flat swamps of Klang and Port Swettenham by more or less ordinary anti-mosquito measures, he was next confronted with the unexpected disappointment of failure in similar crusades in the hill country, where an even better chance of victory had been anticipated. However, Dr. Watson applied himself to the minute investigation of the whole of the mosquito problem, discovered reasons sufficient to account for the failure, and devised a new campaign to pluck victory from defeat. The final results of this are not yet certain, but the success already attained promises very well.

Even if the author had nothing else to his credit as a tropical hygienist but his work during the last fourteen years in the Federated Malay States, he might well claim to be an authority on part, at least, of the malaria problem, and his writings would command respectful attention. But in pursuit of fuller knowledge of his subject Dr. Watson has visited Panama, Guiana, Sumatra, India, and other Eastern countries, and from all of them he has lessons to teach. It is, naturally, of Panama that he has most to say. The wonderful story of the conquest of Nature there by American ingenuity, utilising British research, has been told before, but is by no means stale. We could wish that a paper on tropical hygiene were made compulsory for all candidates for the Indian Civil Service and for Eastern cadetships. Two or three text-books might be indicated, and this should certainly be one of them; Ross's "Prevention of Malaria" and Rubert Boyce's "Yellow Fever and its Prevention" might well be the others.

**Anatomy and Physiology for Nurses.** Second Edition. **A Quiz Book of Nursing.** By A. E. and T. A. Pope. Second Edition. (New York and London: G. P. Putnam's Sons. Price 7s. 6d. net each.)

Both these Transatlantic text-books have reached a second edition, in itself evidence that they have been favourably received. The volume on anatomy and physiology is, in our view, considerably the better of the two. It gives a comprehensive survey of these two subjects, sufficient for the purposes of any examination that a nurse is likely to be called upon to sit for. There are, indeed, occasional excursions into pathology, and perhaps a few more of them might be of advantage. It is, of course, inadvisable in a nursing text-book to go so far as the pathology of tumours; but that of shock, for instance, and that of inflammation would surely be both legitimate and helpful for the understanding of the treatment of those conditions. However, this merely by way of a suggestion. The book, taken all round, is good.

The other book is a deal less admirable. It contains in question and answer form a vast number of paragraphs of instruction in nursing duties. A good many of the answers are far from accurate; some of them reflect views held many years ago but now generally abandoned. There are one thousand questions on nursing proper, fifty on hygiene; fifty on bacteriology, three hundred and fifty on anatomy and physiology, two hundred and fifty on dietetics, and one hundred and fifty on materia medica! After these are chapters on District Nursing, Hospital Construction, and Hospital Accounting. These, be it understood, are formal essays, not "quizzes" of the question-and-answer type. We cannot understand on what principle they are introduced into the text, and for many reasons we do not recommend this book to British nurses.

